# A fast, easy, cost-free method to remove excess dye or drug from small extracellular vesicle solution

**DOI:** 10.1371/journal.pone.0301761

**Published:** 2024-05-08

**Authors:** Ioannis Isaioglou, Gloria Lopez-Madrigal, Jasmeen S. Merzaban

**Affiliations:** 1 Biological and Environmental Science and Engineering Division, Bioscience Program, King Abdullah University of Science and Technology (KAUST), Thuwal, Saudi Arabia; 2 KAUST Smart-Health Initiative, King Abdullah University of Science and Technology, Thuwal, Saudi Arabia; University of Eastern Piedmont: Universita degli Studi del Piemonte Orientale Amedeo Avogadro, ITALY

## Abstract

Tracking small extracellular vesicles (sEVs), such as exosomes, requires staining them with dyes that penetrate their lipid bilayer, a process that leaves excess dye that needs to be mopped up to achieve high specificity. Current methods to remove superfluous dye have limitations, among them that they are time-intensive, carry the risk of losing sample and can require specialized equipment and materials. Here we present a fast, easy-to-use, and cost-free protocol for cleaning excess dye from stained sEV samples by adding their parental cells to the mixture to absorb the extra dye much like sponges do. Since sEVs are considered a next-generation drug delivery system, we further show the success of our approach at removing excess chemotherapeutic drug, daunorubicin, from the sEV solution.

## Introduction

**Fig 1:**
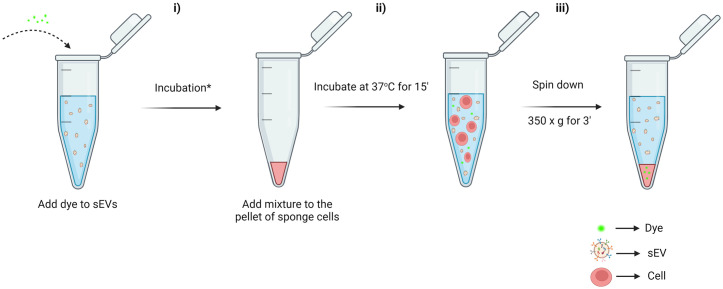
Overview of the protocol to remove the excess dye from the solution containing the stained sEVs. **i)** sEVs incubated with the respective dye/drug. The time and the conditions of this incubation are dependent on the sEV type and the recommendation of the dye/drug supplier. In our case, KG1a-derived sEVs were stained with 2μM of DiO (or 6μM of DNR) for 1 hour at 37°C shaking at 350 rpm [8]. **ii)** The mixture from step **i** is added to a pellet of parental-sponge cells (here KG1a cells) and incubated for 15 minutes at 37°C with shaking at 350 rpm. **iii)** The mixture of sEV with sponge cells were centrifuged for 3 minutes at 350 x g and the resulting supernatant containing the stained sEVs, without excess dye/drug, is ready for downstream applications.

Research on extracellular vesicles has increased dramatically in recent years [1], with a particular focus on understanding how they are taken up by recipient cells and their *in-vivo* organotropic distribution [2–4]. To visualize this uptake and distribution, small extracellular vesicles (sEVs) such as exosomes are stained by using either lipophilic carbocyanine dyes, such as DiO, and/or conjugated antibodies that target their ligands [2]. One key challenge in accomplishing this labelling lies in cleaning the excess dye in the solution due to the nano-scale size of these vesicles, which are at least 100 times smaller than normal cells [5]. While current strategies, such as adding an extra ultracentrifugation step, size exclusion chromatography and resin columns [5], do work, they are time-intensive, carry the risk of losing sample and can require specialized equipment. Here we present a novel, simple method that can, in under 20 minutes, be used to clean the excess dye from the sEVs’ supernatant using basic equipment that can be found in any laboratory (Fig 1). This method has the benefit of saving time, eliminating the cost of additional equipment and supplies and, importantly offering a high yield of final sample.

## Materials and methods

The protocol described in this peer-reviewed article is published on protocols.io dx.doi.org/10.17504/protocols.io.14egn3k4pl5d/v1 and is included for printing as supporting information file 1 with this article. The protocol was published before the new MISEV[6], therefore the word “exosomes” was used instead of “sEVs”.

## Expected results

The provided protocol suggests a fast, free of cost and simple way to remove the excess dye that remains in solution following the staining of sEVs. Here, we provide evidence of the efficiency and the effectiveness of this method. The parental cells, i.e. the cells used to derive the sEVs, used to remove the excess dye will be referred to as “sponge” cells, while the cells used to examine whether the excess dye was totally removed will be referred to as “test” cells. Note that in this example here we used KG1a cells as the parental cells since we are looking at KG1a-derived sEVs from this parental cell type. This can be adjusted depending on which cell type the sEVs are derived from.

**Fig 2:**
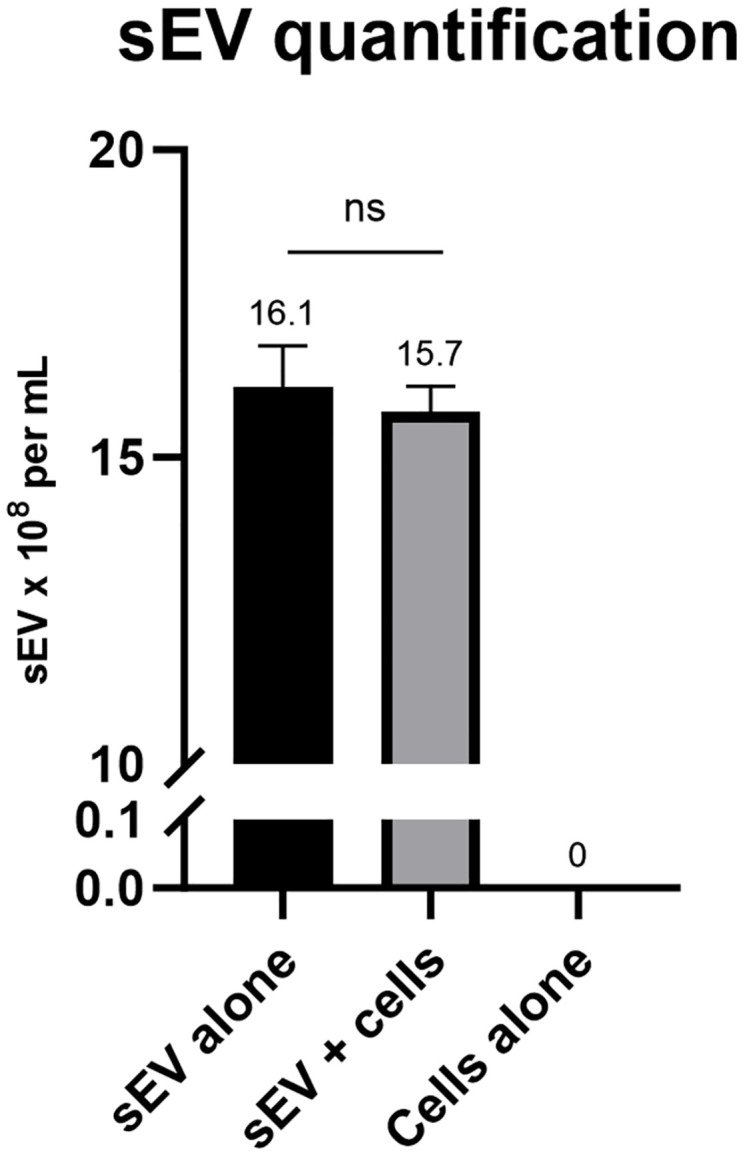
sEV quantification with and without the co-incubation with their parental sponge cells. There was no significant difference between the two samples. On the other hand, cells alone were not able to produce detectable number of sEVs in the same time period (n = 3).

To answer the question of whether any isolated sEVs are lost following the addition of sponge cells to the sEV sample, we isolated and characterized sEVs using standard protocols [7], which were employed as in a previous study from our lab [8]. The isolated sEVs were quantified using the Nanoparticle Tracking Analysis (NTA, S1 Fig) system with and without the 15 minutes of co-incubation with their parental sponge cells. As illustrated in Fig 2, the addition of the sponge cells led to a decrease of about 2% in the concentration of sEVs, compared with sEVs that were not incubated with sponge cells. This decline shows that the sponge cell treatment does not diminish sEV quantity in a significant way. Furthermore, in order to determine whether the sponge-cell approach contributes to nascent sEV production, the same number of sponge cells were placed in PBS buffer of the same volume and, following 15 minutes of incubation, the number of newly produced sEVs was determined by NTA. As illustrated in Fig 2, the incubation time was not enough for the cells to produce detectable numbers of new sEVs (S1 Table).

**Fig 3:**
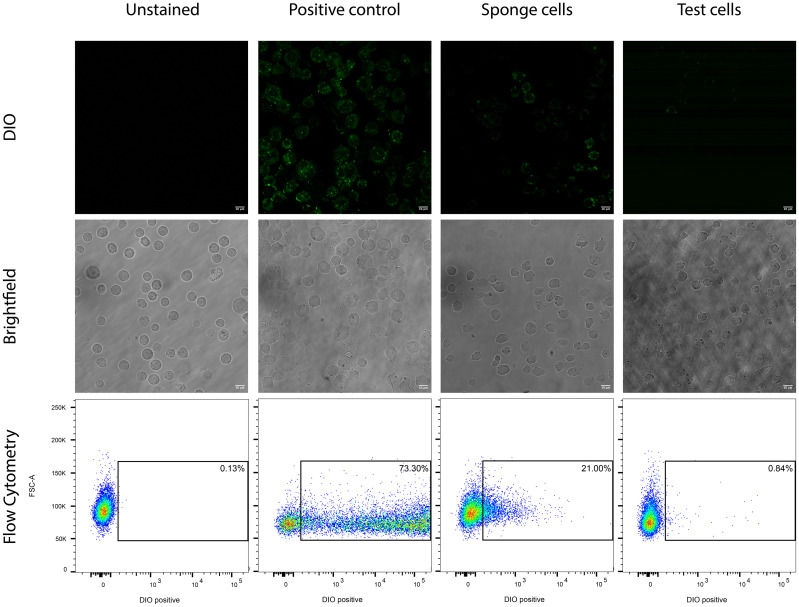
Fluorescent microscopy and Flow Cytometry testing of “sponge” and “test” cells confirmed the successful removal of the excess dye from the supernatant. As indicated, the excess dye was removed as “sponge” cells were partially stained, while the “test” cells had fluorescent signal similar to the unstained cells. Confocal microscopy imaging was performed using an inverted Leica SP8 microscope, while flow cytometry was performed using a BD FACS Canto II cytometer. This is a representative experiment of n = 3 independent experiments.

**Fig 4:**
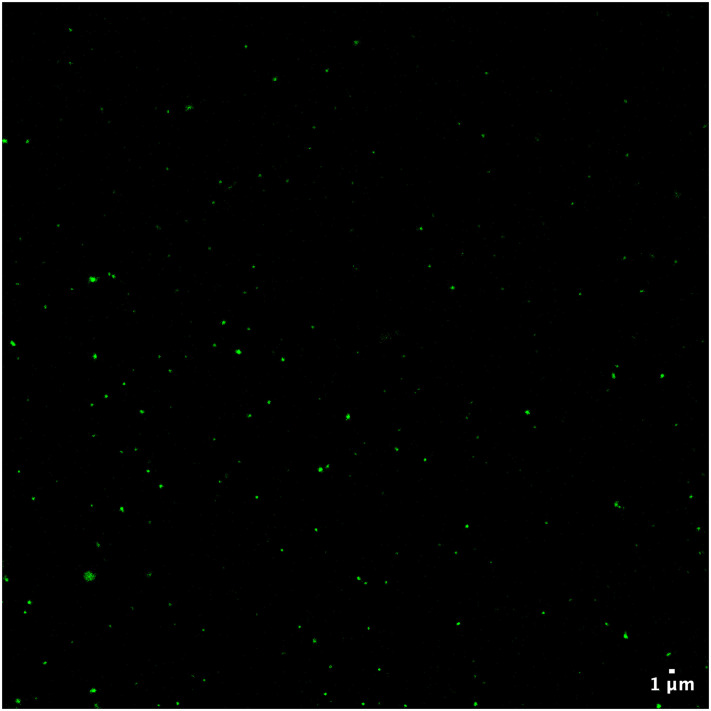
DiO-stained sEVs under fluorescent microscopy. Confocal microscopy imaging was performed using an inverted Leica SP8 microscope. This is a representative image of n = 3 independent experiments.

After confirming that the sponge cell treatment did not affect the quantity or quality of the sEV population, we sought to demonstrate that using sponge cells as a cleaning step is indeed sufficient to remove excess dye. Following the cleaning step, the sponge cells were pelleted down, and the supernatant was removed and added to a new tube containing an equal quantity of parental cells used to “test” whether any excess dye remained in the sample. Following 15 minutes of incubation, the mixture of test cells and sEVs was spun down (Fig 1, step iii), and the resultant supernatant was removed and added to a new tube. The sponge cells and the test cells were then examined using fluorescence microscopy and flow cytometry to determine the amount of dye mopped up by the cells. As evident from Fig 3, while sponge cells stained positive for the DiO dye (i.e. green fluorescence), the test cells did not show any significant uptake of dye, neither by fluorescence microscopy (Fig 3, **top**) nor by flow cytometry (Fig 3, **bottom**). These results indicate that the sponge cells efficiently removed the excess dye since no additional unbound dye was detected in the test cells. As a positive control, a population of parental cells equal in number to the sponge cells used in the main experiment and stained with the same concentration of DiO dye was evaluated. Both assays revealed green fluorescence-stained cells post incubation. The difference in the number of stained cells between the DiO positive control sample and the sponge sample indicates the successful staining of the sEVs by DiO. sEV staining was confirmed by examining the stained sEVs using fluorescence microscopy as previously described in our recent publication[8] (Fig 4).

sEVs can be used as a next generation drug delivery system [9]. Therefore, we wanted to show that our technique has practical application by testing it with a specific drug: daunorubicin (DNR), an anti-tumor activity drug widely used as a treatment for leukemia. DNR possesses fluorescence properties that can be detected by fluorescence microscopy and flow cytometry [10]. We incubated sEVs with DNR and, in order to remove the excess drug from the solution, we applied the same workflow described above. Fluorescence microscopy showed that the sponge cells successfully absorbed the excess DNR, and furthermore, the signal detection by flow cytometry was partially positive compared with control unstained cells. To verify how effectively the sponge cells absorbed the excess DNR from the initial sEV solution, test cells were subsequently added, and, in support of our method, they showed no detectable fluorescence signal in either assay (S2 Fig). These results again demonstrate that the sponge cells efficiently removed the excess drug from the solution. Given the potential of using sEVs for drug delivery, we also aimed to assess how efficiently sEVs took up DNR. To do this, our protocol involved adding to the sEV sample the same concentration of the drug that was added to the positive control cell sample. Following incubation and exposure to sponge cells to mop up extra DNR, we compared the fluorescence signal given off by the sponge cell sample post sEV exposure to the signal given off by the positive control (i.e. cells exposed to DNR alone). We found that sponge cells showed a lower fluorescence signal than the positive control, indicating the successful penetration of DNR to the sEVs.

In conclusion, this protocol provides a fast, easy and low-cost alternative to removing the excess dye that remains during the staining of sEVs such as exosomes, without affecting quantity or quality.

## Supporting information

S1 Fig sEVs with and without incubation with cells. Size distribution of sEVs incubated without cells **(A)** and with cells **(B)**. The size distribution did not show any significant differences. Analysis was done by Nanoparticle Tracking Analysis system. Zeta potential of sEVs incubated without cells **(C)** and with cells **(D)** show no difference in the zeta potential profiles. Western blot analysis **(E)** of sEVs without and with cells incubation showed no difference in the signal of the extracellular vesicle marker CD63. These are representative experiments of three independent replicates.
(TIF)

S2 Fig Fluorescent microscopy and Flow Cytometry testing of “sponge” and “test” cells confirmed the success of removing the excess drug from the supernatant following sEV drug loading. As indicated, the excess drug was removed as “sponge” cells were partially stained, while the “test” cells had fluorescent signal similar to the unstained cells. This is a representative experiment of three independent replicates.
(TIF)

S1 Table Experimental values used for graphs in Fig 2. (DOCX)

S1 File (PDF)
