# Supplementary figures and images for: A fast, easy, cost-free method to remove excess dye or drug from small extracellular vesicle solution
Source: PLoS One. 2024 May 8;19(5):e0301761. doi: 10.1371/journal.pone.0301761 (PMC11078409; doi:10.1371/journal.pone.0301761)

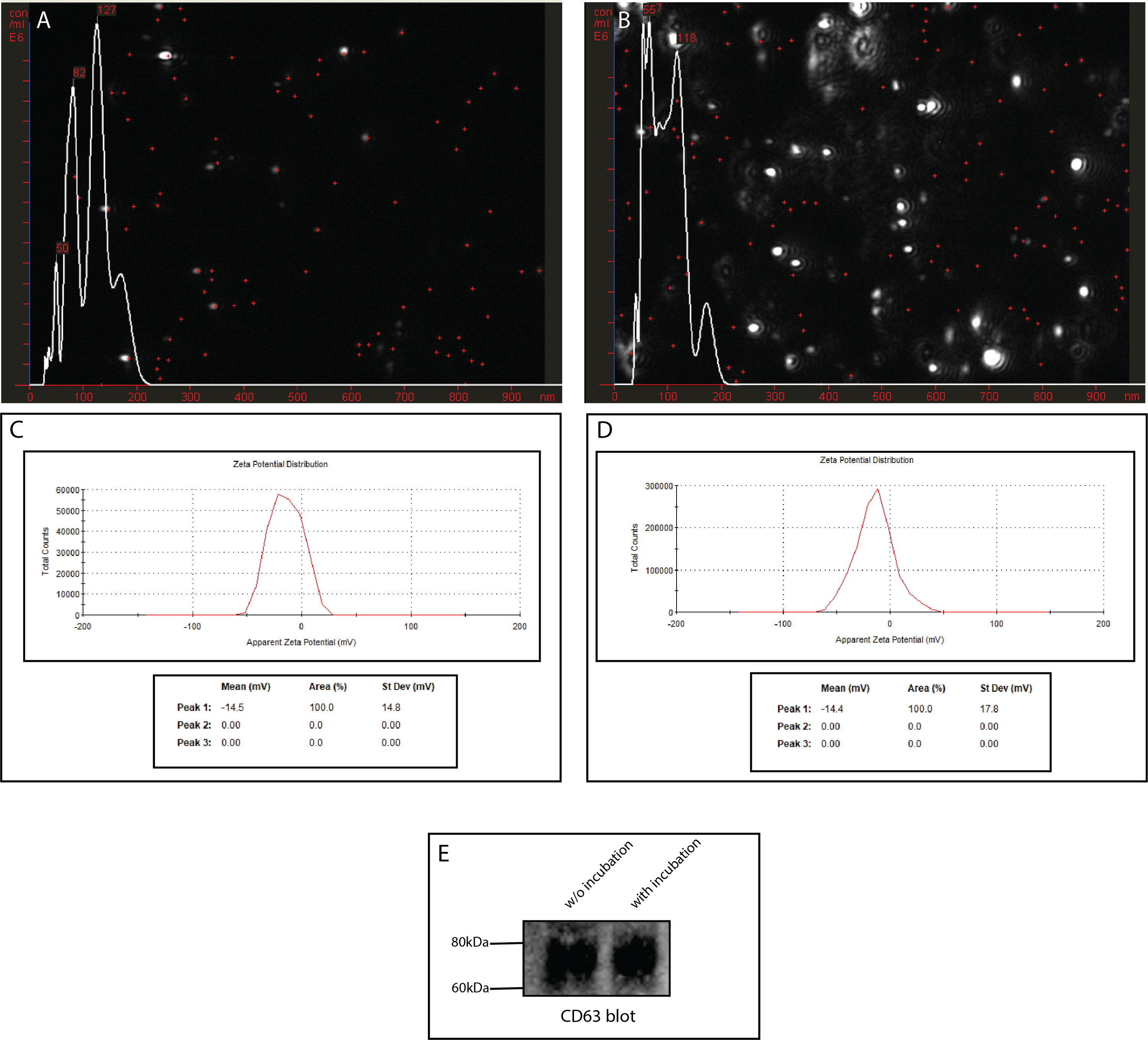

Supplement: S1 Fig — Size distribution of sEVs incubated without cells (A) and with cells (B). The size distribution did not show any significant differences. Analysis was done by Nanoparticle Tracking Analysis system. Zeta potential of sEVs incubated without cells (C) and with cells (D) show no difference in the zeta potential profiles. Western blot analysis (E) of sEVs without and with cells incubation showed no difference in the signal of the extracellular vesicle marker CD63. These are representative experiments of three independent replicates. (TIF) [file pone.0301761.s001.tif]

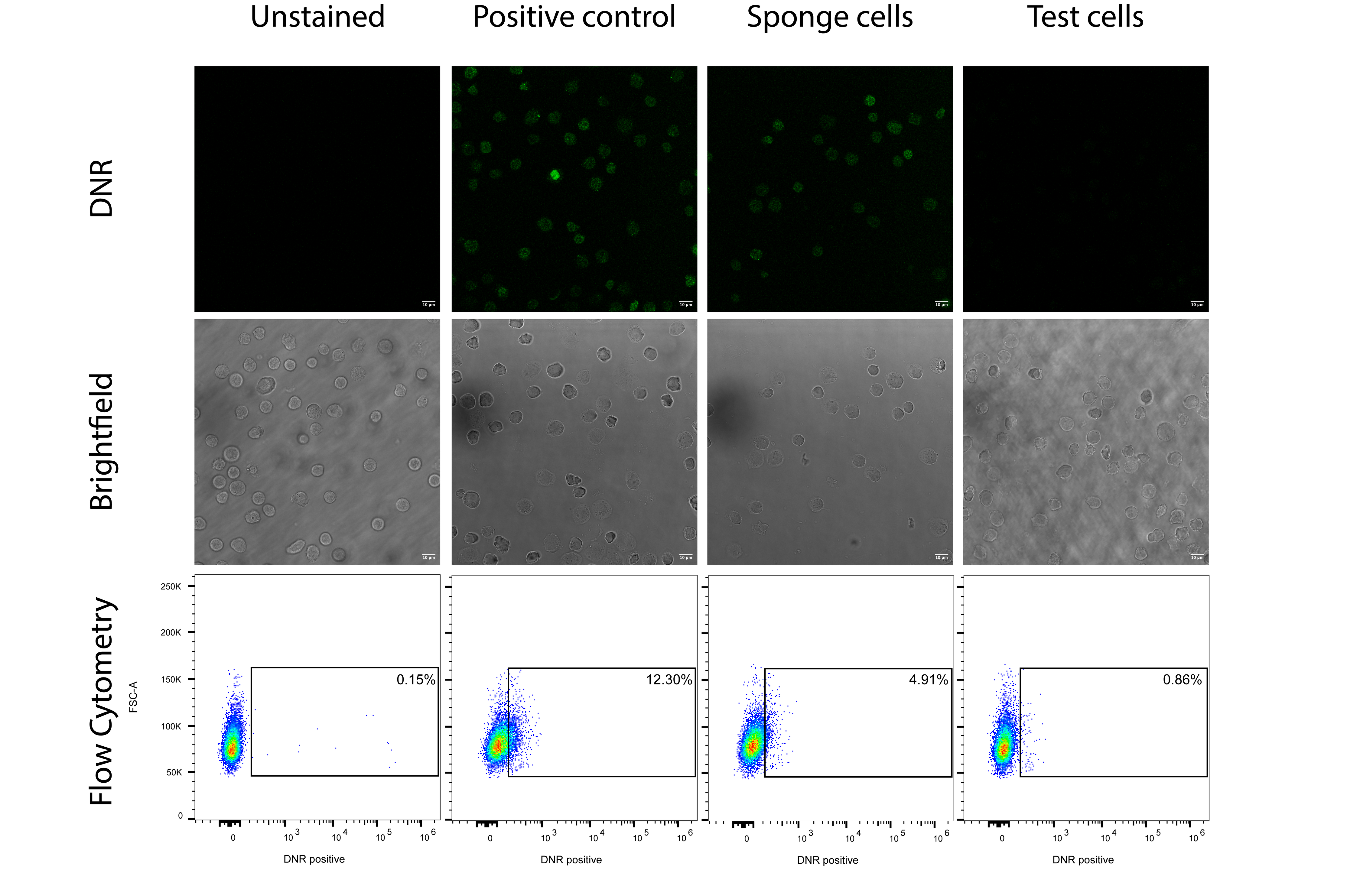

Supplement: S2 Fig — As indicated, the excess drug was removed as “sponge” cells were partially stained, while the “test” cells had fluorescent signal similar to the unstained cells. This is a representative experiment of three independent replicates. (TIF) [file pone.0301761.s002.tif]
