# Supplementary material for: A fast, easy, cost-free method to remove excess dye or drug from small extracellular vesicle solution
Source: PLoS One. 2024 May 8;19(5):e0301761. doi: 10.1371/journal.pone.0301761 (PMC11078409; doi:10.1371/journal.pone.0301761)
Supplement: S1 Table — (DOCX) [file pone.0301761.s003.docx]

|  | sEVs alone | sEVs + cells | Cells alone |
| --- | --- | --- | --- |
| Value_1 | 16.2 | 16.22 | 0 |
| Value_2 | 15.44 | 15.52 | 0 |
| Value_3 | 16.78 | 15.46 | 0 |
|  |  |  |  |
| Mean | 16.14 | 15.73 | 0 |
| St. dev. | 0.6720 | 0.4225 | 0 |
| P-valule (Column B vs C) | 0.4250 |  |  |
