# Supplementary material for: A fast, easy, cost-free method to remove excess dye or drug from small extracellular vesicle solution
Source: PLoS One. 2024 May 8;19(5):e0301761. doi: 10.1371/journal.pone.0301761 (PMC11078409; doi:10.1371/journal.pone.0301761)
Supplement: S1 File — (PDF) [file pone.0301761.s004.pdf]

Apr 19, 2024 Version 1

A fast, easy, cost-free method to remove excess dye or drug from exosome solution V.1

DOI
dx.doi.org/10.17504/protocols.io.14egn3k4pl5d/v1

Ioannis Isaioglou[1], Gloria Lopez-Madrigal[1], Jasmeen Merzaban[1,2]

[1]Bioscience Program, Biological and Environmental Science and Engineering Division, King Abdullah University of Science and Technology (KAUST), Thuwal, Saudi Arabia;
[2]KAUST Smart-Health Initiative, King Abdullah University of Science and Technology, Thuwal, Saudi Arabia

J Jasmeen Merzaban
King Abdullah University of Science and Technology (KAUST)

OPEN ACCESS

DOI: dx.doi.org/10.17504/protocols.io.14egn3k4pl5d/v1

Protocol Citation: Ioannis Isaioglou, Gloria Lopez-Madrigal, Jasmeen Merzaban 2024. A fast, easy, cost-free method to remove excess dye or drug from exosome solution. **protocols.io** **https://dx.doi.org/10.17504/protocols.io.14egn3k4pl5d/v1**

License: This is an open access protocol distributed under the terms of the **Creative Commons Attribution License**, which permits unrestricted use, distribution, and reproduction in any medium, provided the original author and source are credited

Protocol status: Working
We use this protocol and it's working

Created: January 30, 2024

Last Modified: April 19, 2024

Protocol Integer ID: 94703

Funders Acknowledgement:
King Abdullah University of Science and Technology
Grant ID: Faculty Baseline Research Funding Program to Jasmeen Merzaban

Disclaimer

This protocol has been submitted to PLOS ONE and it is under the revision process

Abstract

This protocol details a cost-free method to remove excess dye or drug from exosome solution.

Attachments

pny6cbzb7.pdf

226KB

Guidelines

Tips:

- Cells from step 5 should have signal detectable signal under flow cytometry and/or fluorescent microscopy assay (sponge cells). In case they do not, it is advised to increase the concentration of the dye to ensure that all isolated particles are sufficiently stained.
- Cells from step 9 should have no signal (test cells). If they do, it is advised to optimize the staining conditions. For example, a reduction in the initial amount of dye can be considered. Alternatively, an increase in the number of parental cells used per washing step can also be explored with a caveat that this might reduce the number of exosomes remaining.

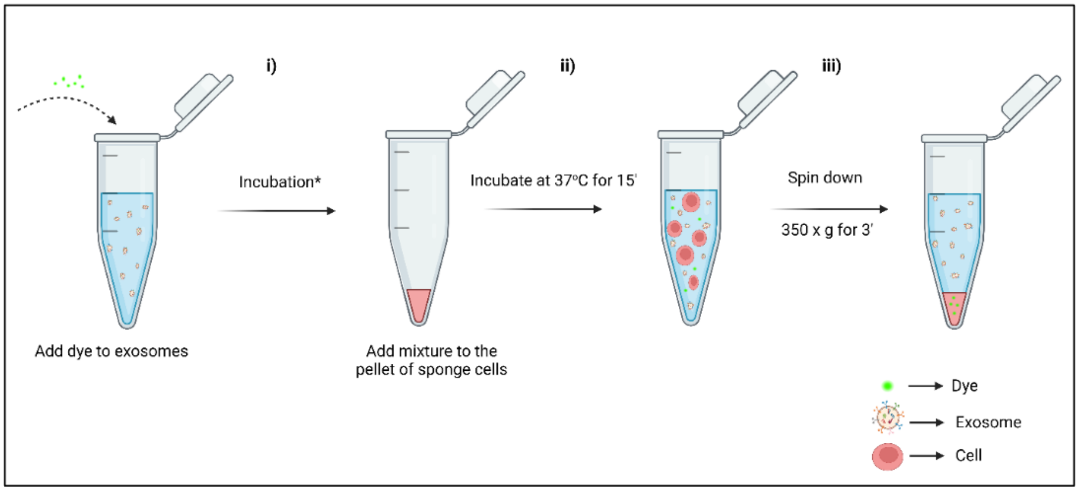

Fig. 1. Overview of the protocol to remove the excess dye from the solution containing the stained exosomes. i) Exosomes incubated with the respective dye/drug. The time and the conditions of this incubation are dependent on
the exosome type and the recommendation of the dye/drug supplier. In our case, KG1a-derived exosomes were stained with 2µM of DiO (or 6µM of DNR) for 1 hour at 37 o C shaking at 350 rpm [1]. ii) The mixture from step i is added to a pellet of parental-sponge cells (here KG1a cells) and incubated for 15 minutes at 37o C with shaking at 350 rpm. iii) The mixture of exosomes with sponge cells were centrifuged for 3 minutes at 350 x g and the resulting
supernatant containing the stained exosomes, without excess dye/drug, is ready for downstream applications. Figure created by BioRender.com.

Materials

Centrifuge (Eppendorf 5415R)

Protocol Steps

36m

1 Collect 0.5 x 10^6 cells (preferably parental cells used for exosome isolation)* and wash them with
phosphate buffered saline (PBS) or alternative buffer.

> **Note**
>
> *Since exosomes derived from their parental cells usually share the same ligands, the likelihood of their ligands interacting is lower.

2 Pellet down the cells (typically 350 x g, Room temperature, 00:03:00). 3m

3 Add the mixture containing the stained exosomes** with the excess dye/drug to the cell pellet.

> **Note**
>
> ** The proposed cleaning protocol has been tested to a range from 100,000 to 1,000,000 exosomes.

4 Resuspend by gently pipetting the cells and leave the mixture shaking at 350 rpm, 37°C, 00:15:00 15m

5 Spin down the mixture so the cells form a pellet using the same parameters as in step 2.

6 Transfer the supernatant into a new clean tube. This supernatant should now contain the stained or loaded exosomes without any excess dye in the buffer solution used.

> **Note**
>
> To optimize the protocol, whenever new types of exosomes/dye are being used, it is advisable to continue with the following steps:

7 Repeat steps 2 through 5: 18m

- Pellet down the cells (typically 350 x g, Room temperature, 00:03:00).
- Add the mixture containing the stained exosomes** with the excess dye/drug to the cell pellet.

- Resuspend by gently pipetting the cells and leave the mixture shaking at 350 rpm, 37°C, 00:15:00
- Spin down the mixture so the cells form a pellet using the same parameters as in step 2.

8 Collect the supernatant which contains the stained exosomes without the excess dye.

9 Resuspend the pellet in PBS buffer (or alternative) and check whether the cells (test cells) were stained. This confirmation step can be verified using fluorescent microscopy or flow cytometry for fluorescent drugs or dyes.

Protocol references

1. **Isaioglou, I., et al.**, CD34(+) HSPCs-derived exosomes contain dynamic cargo and promote their migration through functional binding with the homing receptor E-selectin. Front Cell Dev Biol, 2023. 11: p. 1149912.
